# Clinical application of endoscopic diaphragmotomy and dilation in a congenital duodenal diaphragm

**DOI:** 10.3389/fped.2024.1298748

**Published:** 2024-02-20

**Authors:** Song Sun, Shan Zheng, Jie Wu, Zifei Tang, Chun Shen, Gong Chen, Kuiran Dong

**Affiliations:** ^1^ Surgical Department, Children’s Hospital of Fudan University, Shanghai, China; ^2^ Gastroenterology Department, Children’s Hospital of Fudan University, Shanghai, China

**Keywords:** duodenal diaphragm, endoscopy, diaphragmotomy, balloon dilatation, children

## Abstract

**Background:**

A congenital duodenal diaphragm (CDD) is a rare cause of duodenal obstruction in infants and children. The traditional approach to treatment has been surgical intervention either with excision and duodenoplasty or with bypass through a duodenoduodenostomy, which is invasive and carries the risk of anastomotic leakage.

**Aims:**

To summarize the key points of differential diagnosis and treatment of a CDD under gastroscopy and to evaluate its efficacy and safety.

**Methods:**

The clinical data of patients with a duodenal obstruction diagnosed and treated by gastroscopy in our hospital between January 2019 and December 2022 were retrospectively analyzed. The gastroscopic features of the CDD were summarized. The clinical outcomes of patients with CDD treated by gastroscopy were collected and investigated.

**Results:**

A total of 13 children with a duodenal obstruction underwent a gastroscopic examination and/or treatment, and of these, 10 were diagnosed as having a CDD and 3 were finally diagnosed as having an annular pancreas. A dome-shaped structure during inspiration (9/10) and a guidewire that could be inserted through the opening into the distal lumen (6/10) were the gastroscopic features of the CDD, while the annular pancreas had none of these features. The 10 patients, 4 men and 6 women with the CDD, were treated through endoscopic diaphragmotomy and dilation. The age and weight at operation was 15 days to 7 years (M: 2.25 years) and 2.85–22 kg (M: 13.6 kg), respectively. A total of 11 endoscopic operations were performed in the 10 patients. The time of single operation was 15–55 min (M: 38 min). The patients did not experience complications such as bleeding, pneumoperitoneum, and duodenal papilla injury during the operation. Feeding was started 12–24 h after surgery, and the patients were discharged 2–10 days (M: 7 days) postoperatively. The patients were followed up for 3–36 months (M: 17 months), and none of them had a recurrence of vomiting. However, three showed a recurrence of stenosis in upper gastrointestinal imaging, one of whom underwent a partial diaphragm resection again 2 months after the primary treatment.

**Conclusions:**

A CDD can be confirmed by endoscopy based on its characteristic features. Endoscopic diaphragmotomy and balloon dilation are safe and effective, which can be a new option for minimally invasive treatment of a CDD.

## 1 Introduction

A congenital duodenal diaphragm (CDD), also referred to as duodenal membranous atresia or a duodenal web, is a rare pediatric surgical condition with an incidence of 1–2/10,000 (1, 2) and commonly manifests as bile-stained vomiting. Typically, the diaphragm exhibits openings that communicate with the distal lumen. The degree of vomiting and the time of onset vary with the size and patency of the openings. Infants with smaller openings typically experience severe vomiting during their initial feeding after birth, necessitating early neonatal surgical intervention. Conversely, those with larger openings may present intermittent vomiting symptoms and delay seeking medical attention for months or even years following birth. The prognosis for a CDD postsurgery is generally favorable in the absence of concurrent severe malformations.

The surgical approach for treating a CDD has evolved from open surgery to a laparoscopic side-to-side/rhomboid anastomosis of the duodenum or duodenotomy and diaphragmectom (3–5). However, both approaches involve invasive procedures such as entering the peritoneal cavity, incising, and reanastomosing the duodenum, which pose potential risks of infection, anastomotic leakage, intestinal adhesion, and obstruction (3). Since the 1980s (6–8), an endoscopic laser ablation of an adult duodenal diaphragm has been reported as an effective and less invasive alternative (9–12). Over the last 30 years, sporadic reports on an endoscopic treatment of CDD in children have emerged with advancements in endoscopic techniques and equipment. In 1989, Okamatsu et al. successfully treated a 2-month-old boy with Down syndrome using endoscopic therapy (9). Kay et al. first applied this approach to four neonates in 1992 but achieved successful outcomes only in one neonate (13). In the last two decades, there has been a gradual increase in reports of endoscopic treatment (10–12). Some of the reported methods are laser ablation (6, 8), balloon dilation (12, 14–16), diaphragmotomy (15, 17), and diaphragmectomy (18–20). Although most reports have shown positive outcomes, except for a few cases of perforation and restenosis (13, 15, 21, 22), because of their type, that is, being case reports rather than systematic studies, it is still premature to draw definitive conclusions regarding the safety and efficacy of the aforementioned methods.

Herein, we conducted a retrospective study analyzing clinical data from children with a CDD who underwent endoscopy at our hospital, aiming to summarize key points related to the diagnosis and treatment of a CDD through endoscopy, while evaluating its safety and efficacy.

## 2 Methods

Patients suspected of having a CDD and who underwent endoscopic diagnosis and treatment in Children's Hospital of Fudan University from January 2019 to December 2022 were included in this study. The clinical data regarding hospitalization and postoperative follow-up were retrospectively collected and analyzed. The endoscopic appearance of the CDD and the technical points of endoscopic treatment were summarized and presented.

This study was approved by the Ethics Commission of Children's Hospital of Fudan University.

### 2.1 Identification of the CDD and determination of the diaphragm boundaries under gastroscopy

**Figure 1:**
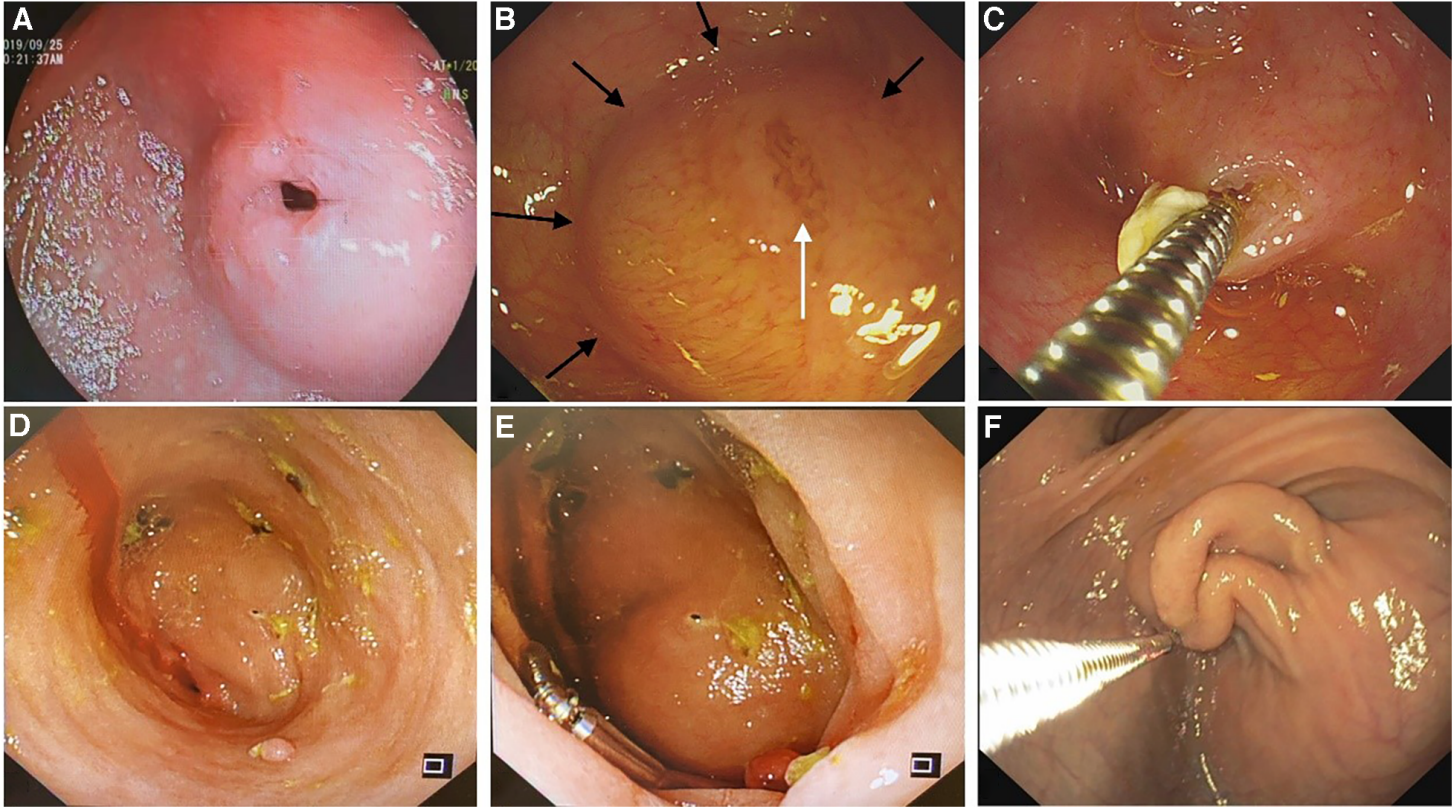
Various gastroscopic features of a duodenal diaphragm. (**A**) A diaphragm with a well-defined opening. (**B**) A diaphragm with a mucosal fissure (white arrow): a dome-shaped structure can be seen during inspiration, and the edge of the diaphragm can be judged according to the boundary of the dome structure (black arrows). (**C**) A metal guidewire is inserted into the distal intestine through the mucosal fissure in the diaphragm. (**D**) The diaphragm appears as a blind end without any visible opening. (**E**) A dome-shaped structure can be seen during inspiration when the diaphragm appears as a blind end. (**F**) The diaphragm prolapses proximally during inspiration to form a sleeve-like structure in an older patient.

After anesthesia induction, a routine gastroscopic examination was performed at the site of obstruction to assess whether there was a visible opening or its location. When suctioned at the proximal end, a dome-shaped structure protruded proximally or a mucosa sleeve prolapsed into the proximal lumen. If a visible opening or mucosal fissure was present, an attempt was made to insert a metal guide wire into the distal lumen, so that the diaphragm and the edge of the diaphragm could be observed with the assistance of the guide wire. The presence of the diaphragm and its boundary can be confirmed through these gastroscopic features (Figure 1). If there was a suspected opening but a metal guide wire could not be passed through it, along with a collapsed bowel without dome-shaped structures or intraluminal mucosa sleeves during inspiration, the diagnosis of a CDD was excluded and the endoscopic operation was terminated and converted to a transabdominal surgery.

### 2.2 An endoscopic incision and balloon dilation of the diaphragm

**Figure 2:**
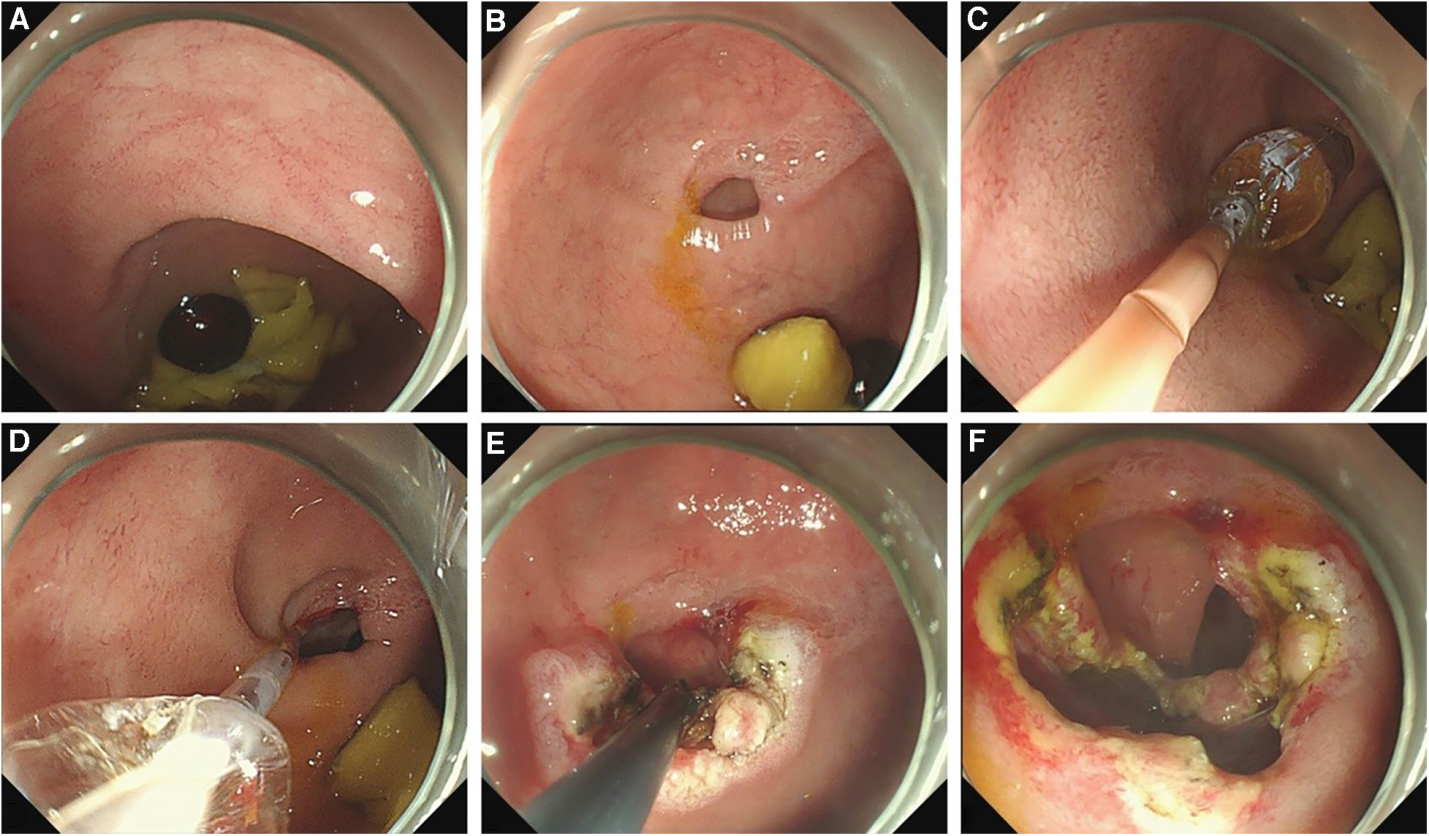
The procedure of an endoscopic treatment of a duodenal diaphragm with a well-defined opening. (**A**) A food debris obstruction at the site of the duodenal diaphragm is observed by gastroscopy. (**B**) An eccentric opening can be seen after the obstructed food residue is removed. (**C**) A balloon is placed through the opening to enlarge the opening. (**D**) After dilation, the distal bowel wall can be seen through the expanded opening to determine the direction and scope of the incision. (**E**) The diaphragm is incised radially to the opposite direction of the bowel wall. (**F**) A part of the diaphragm is removed and the opening is enlarged further.

The diaphragm was incised from the opening on the diaphragm or the top center of the dome structure with a hook knife, to enable the distal cavity to directly communicate with the proximal cavity. The incision could be made radially to the opposite side of the eccentric direction of the opening. After incision, a snare could be used to remove a part of the redundant septal mucosa. Balloon dilatation (12–16 mm) was performed before or after incision in patients in whom the duodenal papilla could not be identified (Figure 2). After incision and dilation, the enlarged opening could be accessed with a 9.3 mm gastroscope. At this stage, the distal intestine and duodenal papilla should be detected to ensure no injuries. The diaphragm in some patients was quite thick, and it needed to be incised in two separate layers, the oral side layer and the anal side layer, to pierce the proximal and distal lumen (Supplementary Material Video S1).

## 3 Results

A total of 13 consecutive patients with a suspected CDD underwent gastroscopy at Children's Hospital of Fudan University between January 2019 and December 2022. Among them, 10 patients, 4 males and 6 females, were confirmed to have a CDD under gastroscopy. The remaining three were diagnosed with an annular pancreas and they subsequently underwent a laparoscopic duodenal diamond-shaped anastomosis. The age of the 10 patients with the CDD who underwent endoscopic treatment ranged from 15 days to 7 years, with a median age of 2.25 years, and their weights ranged from 2.85 to 22 kg with a median weight of 13.6 kg. Two patients were treated as newborns at the ages of 15 and 21 days, respectively. The chief complaints among the patients with the CDD were intermittent vomiting since the neonatal period (4/10) or after 6 months of age (6/10). One patient had undergone a diamond-shaped anastomosis of the duodenum in a local hospital during the neonatal period; one underwent a thoracoscopic repair for type IIIa esophageal atresia 5 days after birth and an esophageal hiatal hernia repair 1 year later; another underwent patent ductus arteriosus (PDA) ligation and atrial septal defect repair at 2 years of age. None of the remaining 7 patients out of the 10 had a previous surgical history. Among nine patients who received an upper gastrointestinal imaging (UGI) examination, five had an obstruction at the descending part, while four had an obstruction at the horizontal part of the duodenum (Table 1).

**Table 1:** The clinical data of 10 patients with a CDD who underwent endoscopic treatment.

No.	Age	Gender	Weight (kg)	Operation history	UGI	Endoscopic treatment	Follow-up time (months)	Clinical outcome
1	7 years	F	22	Laparoscopic rhomboid anastomosis	Incomplete obstruction of the descending duodenum	Diaphragmotomy	36	Asymptomatic stenosis
2^a^	5 years	M	15	Ligation of PDA, repair of ASD	—	Diaphragmotomy and partial diaphragmectomy	30	Complete resolution of symptoms Patency of passage in UGI
3	9 months	F	7.2	—	Incomplete obstruction of the descending duodenum	Diaphragmotomy	26	Asymptomatic stenosis
4	21 days	F	3.09	—	Incomplete obstruction of the horizontal part of the duodenum	Diaphragmotomy and balloon dilation (12 mm)	24	Complete resolution of symptoms Patency of passage in UGI
5	2 years	M	13.2	—	Incomplete obstruction of the horizontal part of the duodenum	Diaphragmotomy	17	Asymptomatic stenosis^b^
6	5.75 years	F	14	Thoracoscopic repair of EA, Laparoscopic fundoplication	Incomplete obstruction of the descending duodenum	Diaphragmotomy and balloon dilation (16 mm)	17	Complete resolution of symptoms Patency of passage in UGI
7	3.25 years	F	15	—	Incomplete obstruction of the horizontal part of the duodenum	Diaphragmotomy and balloon dilation (16 mm)	12	Complete resolution of symptoms Patency of passage in UGI
8	15 days	M	2.85	—	Obstruction of the horizontal part of the duodenum	Diaphragmotomy and balloon dilation (12 mm)	5	Complete resolution of symptoms Patency of passage in UGI
9	1.5 years	F	10.5	—	Incomplete obstruction of the descending duodenum	Diaphragmotomy and partial diaphragmectomy	3	Complete resolution of symptoms Patency of passage in UGI
10	2.5 years	M	14	—	Incomplete obstruction of the descending duodenum	Diaphragmotomy	3	Complete resolution of symptoms Patency of passage in UGI

Y, year; M, month; D, Day; F, female; M, male; ASD, atrial septal defect; EA, esophageal atresia.

^a^ This patient was diagnosed by gastroscopy performed in the gastroenterology clinic.

^b^ A UGI at follow-up showed a recurrence of duodenal stenosis and duodenal bulb dilatation with antiperistalsis in this boy. Despite being asymptomatic, he underwent a second endoscopic diaphragmotomy and a partial diaphragmectomy 1 month after the primary treatment.

**Figure 3:**
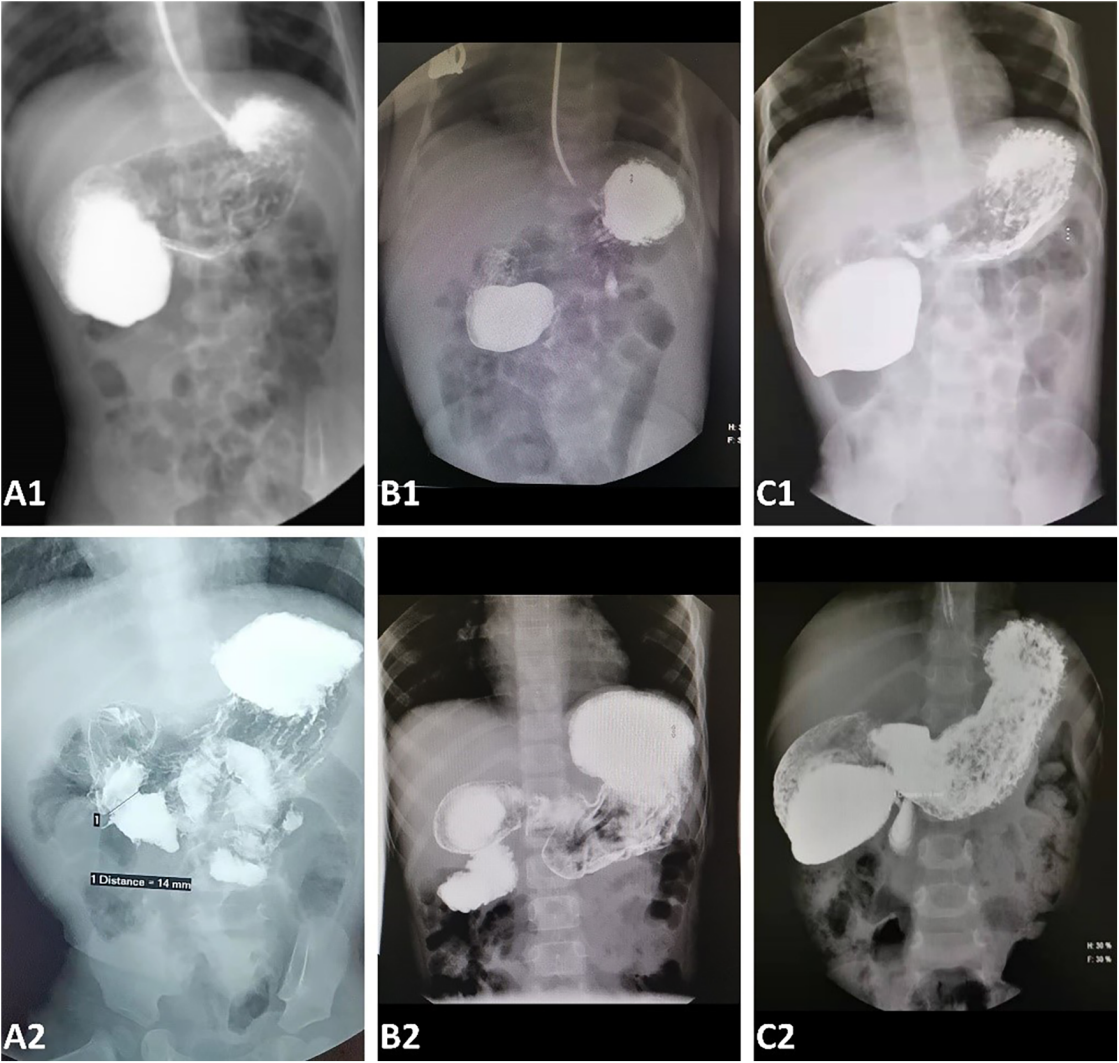
An upper gastrointestinal contrast before (**A1**–**C1**) and 1 month after endoscopic treatment (**A2**–**C2**). (**A**) An upper gastrointestinal contrast 1 month after the operation (**A2**) showed that the duodenal obstruction (**A1)** disappeared and there was no recurrence of stenosis. (**B**) A duodenal obstruction before endoscopic treatment (**B1**) was almost completely removed, but a mild asymptomatic stenosis persisted (**B2**). (**C**) The duodenal stenosis recurred at a diameter of 3 mm. An endoscopic partial diaphragmectomy was performed for this patient 1 month after the primary treatment.

Eleven endoscopic treatments were performed on the 10 patients with the CDD. During the primary treatment, four patients had undergone an endoscopic diaphragmotomy/partial resection and balloon dilation, while six had undergone diaphragmotomy/resection alone. The operation time was 15–55 min with a median time of 38 min. All duodenal diaphragms were incised or dilated to more than 1 cm. There was no intraoperative bleeding, duodenal perforation, pneumoperitoneum, or duodenal papilla injury. All treated patients were started on a liquid diet within 24 h postsurgery, and normal full-volume oral feeding was resumed within 2–5 days (median time 3 days). Prophylactic antibiotics were not administered to them during the perioperative period, while therapeutic antibiotics were prescribed for two patients who experienced postoperative fever and elevated C-reactive protein levels within 48 h. Three of the older patients among them reported abdominal discomfort within 72 h after the operation, whereas no complaints were reported by other patients and their caregivers. A transient elevation of serum amylase (<2 times the reference value) was observed in all three detected patients after the surgery, which resolved within 72 h. The median length of postoperative hospital stay was 7 days (range: 2–10 days). The patients were followed up for 3–36 months (median time 17 months), and no vomiting related to duodenal obstruction recurred during the follow-up periods. All patients underwent a routine UGI at 1 month and again at 3–6 months postoperatively. Asymptomatic stenosis was identified in three patients (Figure 3). One of them underwent an endoscopic partial diaphragmectomy because of persistent abnormal UGI findings 1 month after primary treatment (Figure 3C), resulting in a complete resolution of symptoms and restoration of passage patency on subsequent UGI examination.

## 4 Discussion

As our case series demonstrates, endoscopic diaphragmotomy and dilation appear to represent a minimally invasive and precise management approach for treating a CDD. An accurate identification of a CDD under endoscopy is the prerequisite for safe and effective endoscopic treatment. The appearance of a duodenal diaphragm on preoperative UGI is similar to that of an annular pancreas, making it difficult to distinguish between the two. The following features should be noted for identifying a diaphragm (Figure 1): The proximal end of the obstruction may present as a blind end with or without depression or fissure in the mucosa (10, 19, 23), or as a stenosis with a well-defined opening under gastroscopy (19, 20, 24–26). During inspiration with the gastroscope, the diaphragm forms a dome-shaped structure (19, 20), and its boundary with the bowel wall can be recognized. In older children, due to long-term proximal pressure, the diaphragm becomes elongated and hypertrophic, presenting as a sleeve-like structure prolapse proximally during inspiration. For those in whom there is an opening in the diaphragm, the insertion of a balloon or guide wire allows a visualization of both the distal intestinal lumen and the boundary by a pull back of the balloon or with the assistance of the guide wire. However, in this study, none of our patients with annular pancreas displayed the aforementioned endoscopic findings. Suspected openings could not accommodate guidewire insertion possibly due to extreme distortion caused by dilatation in the proximal bowel and fixation of the annular pancreas at the obstruction site. The diagnosis of a CDD was excluded in these three patients who were confirmed to have annular pancreas by laparoscopic surgery.

Although no injury occurred to the duodenal papilla in previously reported patient cases nor in the patients in this study, ensuring the avoidance of such injury during endoscopic treatment remains a significant concern. The left side of the duodenum is constrained by structures such as the mesentery and pancreaticobiliary duct, making it less flexible than the right side, which can be easily expanded and extended. Consequently, diaphragmatic openings are predominantly eccentrically distributed close to the papilla's side (left side) (17, 19). If the duodenal papilla is visible in gastroscopy, the duodenal papilla can be avoided by incising the diaphragm from the opening to the opposite side of the papilla (10, 11, 17, 20) (Figure 2). Nose et al. (17, 18, 27) employed a balloon-assisted traction on the diaphragm toward its proximal end to determine its extent before performing incision or ablation, thereby preventing injury to both the duodenal wall and the papilla. In addition, using balloon dilation instead of an electrosurgical device may serve as an alternative approach for avoiding injury to the duodenal papilla (12, 18). It has been reported that the opening was dilated using a balloon until the distal cavity could be directly observed under gastroscopy, and then the diaphragm was incised using a hook knife, which helped avoid intestinal wall and duodenal papilla injury (18). In approximately 50% of patients who underwent endoscopic treatment, a visualization of their duodenal papillae was not feasible (12, 22). The diaphragm opening was not obvious under gastroscopy in some patients either (23). In such patients, the diaphragm could be incised laterally from the center and then enlarged with balloon dilation, thus reducing the risk of duodenal papilla injury (20).

Stenosis recurrence is the most prevalent complication reported thus far (15, 17, 21, 22), which may primarily be attributed to inadequate diaphragmotomy and dilation or a rehealing of the diaphragm (21). Currently, there is no standard for prescribing the diameter of the diaphragmotomy or dilatation in children across different age groups. Previous studies suggest that 13.5–14 mm balloons can be utilized in newborns and infants within one month (22, 28), while 15 mm balloons are suitable for infants under 3 years old (29), and 18 mm balloons are appropriate for children aged 3–9 years (14). These reports serve as a valuable reference for balloon dilatation in pediatric patients of varying ages. A T-shaped (7–9) or radial (11) incision may be superior to a linear incision in avoiding rehealing. A further enlargement of the opening by removing a part of the diaphragm by snare or biopsy forceps after diaphragmotomy can potentially prove effective (27, 28) (Figure 2). Symptomatic restenosis can be managed through a re-endoscopic incision or dilatation, as well as transabdominal surgery if necessary (14, 15). However, currently, no report is available on whether asymptomatic stenosis requires a reintervention (17). Among our three patients with asymptomatic stenosis, one underwent a second endoscopic treatment (Figure 3). During this subsequent procedure, it was observed that the original incision site had partially healed, and a significant stricture recurred, with food incarceration at the proximal of the stricture. The remaining two patients were followed up for over 6 months, and they did not exhibit any abnormal symptoms (Figure 3). Therefore, long-term follow-up is required to determine whether intervention is warranted for asymptomatic stenosis.

Although this study is the largest among the case series reported to date, its sample size is still not large enough. The longest period of follow-up in our study was 3 years, and therefore, the long-term prognosis of the treatment modality mentioned in this study is unknown. Moreover, this study is a retrospective one and lacks prospective controlled trials.

In conclusion, endoscopic diaphragmotomy and dilation represent a minimally invasive and precise management approach for treating a CDD. This technique offers a safe and effective alternative with enhanced postoperative recovery rates and reduced risks of anastomotic leakage and abdominal infection. Nevertheless, a greater use of this method is required to mitigate potential recurrence risks as well as minimize the occurrence of side injuries (15).

## Data Availability

The original contributions presented in the study are included in the article/Supplementary Material, and further inquiries can be directed to the corresponding authors.

## References

[1] Takahashi D Hiroma T Takamizawa S Nakamura T. Population-based study of esophageal and small intestinal atresia/stenosis. Pediatr Int. (2014) 56(6):838–44. 10.1111/ped.12359 24730728

[2] Hemming V Rankin J. Small intestinal atresia in a defined population: occurrence, prenatal diagnosis and survival. Prenat Diagn. (2007) 27(13):1205–11. 10.1002/pd.1886 17994616

[3] Holler AS Muensterer OJ Martynov I Gianicolo EA Lacher M Zimmermann P. Duodenal atresia repair using a miniature stapler compared to laparoscopic hand-sewn and open technique. J Laparoendosc Adv Surg Tech A. (2019) 29(10):1216–22. 10.1089/lap.2019.0057 31150305

[4] Chiarenza SF Bucci V Conighi ML Zolpi E Costa L Fasoli L Duodenal atresia: open versus MIS repair-analysis of our experience over the last 12 years. Biomed Res Int. (2017) 2017:4585360. 10.1155/2017/4585360 28326320 PMC5343219

[5] Okata Y Bitoh Y Miyauchi H Aida Y Nakatani T. Laparoscopic reconstruction of double duodenal atresia in a neonate: novel procedure. Pediatr Int. (2019) 61(5):513–5. 10.1111/ped.13823 31099096

[6] Gertsch P Mosimann R. Endoscopic laser treatment of a congenital duodenal diaphragm in an adult. Gastrointest Endosc. (1984) 30(4):253–4. 10.1016/S0016-5107(84)72399-6 6479548

[7] Jex RK Hughes RJ. Endoscopic management of duodenal diaphragm in the adult. Gastrointest Endosc. (1986) 32(6):416–9. 10.1016/S0016-5107(86)71927-5 3803843

[8] Al-Kawas FH. Management of a duodenal web by endoscopic laser therapy. Gastrointest Endosc. (1989) 35(2):113–5. 10.1016/S0016-5107(89)72723-1 2714593

[9] Okamatsu T Arai K Yatsuzuka M Ishikawa M Matsumura M Okamoto S Endoscopic membranectomy for congenital duodenal stenosis in an infant. J Pediatr Surg. (1989) 24(4):367–8. 10.1016/S0022-3468(89)80271-4 2732877

[10] Wood LS Kastenberg Z Sinclair T Chao S Wall JK. Endoscopic division of duodenal web causing near obstruction in 2-year-old with trisomy 21. J Laparoendosc Adv Surg Tech A. (2016) 26(5):413–7. 10.1089/lap.2015.0462 26913816

[11] Bleve C Costa L Bertoncello V Ferrara F Zolpi E Chiarenza SF. Endoscopic resection of a duodenal web in an 11-month-old infant with multiple malformations. Endoscopy. (2015) 47(Suppl 1 UCTN):E210–1. 10.1055/s-0034-1391777 26062152

[12] Huang MH Bian HQ Liang C Wei WQ Duan XF Yang J. Gastroscopic treatment of membranous duodenal stenosis in infants and children: report of 6 cases. J Pediatr Surg. (2015) 50(3):413–6. 10.1016/j.jpedsurg.2014.10.045 25746699

[13] Kay GA Lobe TE Custer MD Hollabaugh RS. Endoscopic laser ablation of obstructing congenital duodenal webs in the newborn: a case report of limited success with criteria for patient selection. J Pediatr Surg. (1992) 27(3):279–81. 10.1016/0022-3468(92)90846-Y 1500998

[14] Poddar U Jain V Yachha SK Srivastava A. Congenital duodenal web: successful management with endoscopic dilatation. Endosc Int Open. (2016) 4(3):E238–41. 10.1055/s-0041-110955 27004237 PMC4798844

[15] Goring J Isoldi S Sharma S Torroni F Marven S De Angelis P Natural orifice endoluminal technique (NOEL) for the management of congenital duodenal membranes. J Pediatr Surg. (2020) 55(2):282–5. 10.1016/j.jpedsurg.2019.10.025 31839373

[16] Wu L Jia G Hu Y Zhu L Wang S. A rare case of duodenal diaphragm in an adult during ERCP treatment for choledocholithiasis. BMC Surg. (2020) 20(1):273. 10.1186/s12893-020-00934-1 33160346 PMC7648286

[17] Blanco-Rodriguez G Penchyna-Grub J Porras-Hernandez JD Trujillo-Ponce A. Transluminal endoscopic electrosurgical incision of fenestrated duodenal membranes. Pediatr Surg Int. (2008) 24(6):711–4. 10.1007/s00383-008-2142-8 18414879

[18] Bittencourt PF Malheiros RS Ferreira AR Carvalho SD Filho PP Tatsuo ES Endoscopic treatment of congenital duodenal membrane. Gastrointest Endosc. (2012) 76(6):1273–5. 10.1016/j.gie.2012.01.022 22421495

[19] Fukuda SI Ichida K Kitagawa Y Nakano K Tomohito C Yoshimura D An adult case of congenital duodenal diaphragm that was successfully treated by endoscopic resection using a grasping-type scissor forceps. DEN Open. (2022) 2(1):e93. 10.1002/deo2.93 35310724 PMC8824441

[20] Beeks A Gosche J Giles H Nowicki M. Endoscopic dilation and partial resection of a duodenal web in an infant. J Pediatr Gastroenterol Nutr. (2009) 48(3):378–81. 10.1097/MPG.0b013e31818c600f 19274797

[21] Barabino A Arrigo S Gandullia P Vignola S. Duodenal web: complications and failure of endoscopic treatment. Gastrointest Endosc. (2012) 75(5):1123–4. 10.1016/j.gie.2011.12.036 22520887

[22] Kong C Li L Dong N Li X Zhang Y. Endoscopic operation in the treatment of congenital duodenal membranous stenosis. Zhonghua Wei Chang Wai Ke Za Zhi. (2015) 18(8):801–3. 26303690

[23] Nicholson MR Acra SA Chung DH Rosen MJ. Endoscopic diagnosis of duodenal stenosis in a 5-month-old male infant. Clin Endosc. (2014) 47(6):568–70. 10.5946/ce.2014.47.6.568 25505725 PMC4260107

[24] Lee SS Hwang ST Jang NG Tchah H Choi DY Kim HY A case of congenital duodenal web causing duodenal stenosis in a Down syndrome child: endoscopic resection with an insulated-tip knife. Gut Liver. (2011) 5(1):105–9. 10.5009/gnl.2011.5.1.105 21461083 PMC3065085

[25] Kamal N Hogan C Midulla PS Dimaio CJ. Successful endoscopic needle-knife catheter membranotomy of a congenital duodenal web. J Pediatr Gastroenterol Nutr. (2015) 61(5):e22–3. 10.1097/MPG.0000000000000323 26505961

[26] Luffy R Troendle DM. Endoscopic management of duodenal web. J Pediatr Gastroenterol Nutr. (2019) 69(4):e117. 10.1097/MPG.0000000000002250 30628986

[27] Nose S Kubota A Kawahara H Okuyama H Oue T Tazuke Y Endoscopic membranectomy with a high-frequency-wave snare/cutter for membranous stenosis in the upper gastrointestinal tract. J Pediatr Surg. (2005) 40(9):1486–8. 10.1016/j.jpedsurg.2005.05.053 16150355

[28] van Rijn RR van Lienden KP Fortuna TL D’Alessandro LC Connolly B Chait PG. Membranous duodenal stenosis: initial experience with balloon dilatation in four children. Eur J Radiol. (2006) 59(1):29–32. 10.1016/j.ejrad.2006.03.015 16621398

[29] Mochizuki K Obatake M Kosaka T Tokunaga T Eguchi S Kanematsu T. Endoscopic balloon dilatation for congenital membranous stenosis in the jejunum in an infant. Pediatr Surg Int. (2011) 27(1):91–3. 10.1007/s00383-010-2714-2 20848289

